# Anomalies of Brillouin Light Scattering in Selected Perovskite Relaxor Ferroelectric Crystals

**DOI:** 10.3390/ma16020605

**Published:** 2023-01-08

**Authors:** Venkatasubramanian Sivasubramanian, Sarveswaran Ganesamoorthy, Seiji Kojima

**Affiliations:** 1 Condensed Matter Physics Division, Indira Gandhi Centre for Atomic Research, Kalpakkam 603102, India; 2 Homi Bhabha National Institute, Mumbai 400094, India; 3 Division of Materials Science, University of Tsukuba, Tsukuba 305-8573, Japan

**Keywords:** disordered ferroelectrics, perovskites, polar nanoregions, Brillouin scattering, acoustic phonon, relaxation

## Abstract

Compositionally disordered perovskite compounds have been one of the exotic topics of research during the past several years. Colossal piezoelectric and electrostrictive effects have been observed in disordered perovskite ferroelectric materials. The key ingredient in the physical behavior of disordered perovskites is the nucleation and growth of the local dipolar regions called polar nanoregions (PNRs). PNRs begin to nucleate far above the temperature of the dielectric maximum *T_m_* and exhibit varied relaxation behavior with temperature. The evidence for the existence of various stages in the relaxation dynamics of PNRs was revealed through the study of the temperature evolution of optical phonons by Raman scattering. The quasi-static regime of PNRs is characterized by the strong coupling between the local polarization and strain with the local structural phase transition and the critical slowing of the relaxation time. Strong anomalies in the frequency and the width of the acoustic phonons, and emergence of the central peak in the quasi-static region of the relaxation dynamics of PNRs have been observed through Brillouin scattering studies. In this review, we discuss the anomalies observed in Brillouin scattering in selected disordered perovskite ferroelectrics crystals such as Pb(Mg_1/3_Ta_2/3_)O_3,_ Pb(Sc_1/2_Ta_1/2_)O_3_, 0.65PIN-0.35PT and Sr_0.97_Ca_0.03_TiO_3_ to understand dynamical behavior of PNRs.

## 1. Introduction

The ABO_3_ perovskite-type ferroelectric oxides have been extensively studied over several decades both from the fundamental and applications point of view. The prototype ferroelectrics such as BaTiO_3_ and KNbO_3_undergo a sequential structural phase transition from initial cubic phase to final rhombohedral phase, involving softening of zone-center optical phonon modes in each phase. On the other hand, PbTiO_3_exhibits a single-phase transition from cubic to tetragonal ferroelectric phase and is regarded as a text book example of the soft-mode-driven phase transition. A ferroelastic transition at *T_tr_* = 110 K driven by the softening of R_25_ zone boundary phonon mode was observed in SrTiO_3_, while KTaO_3_ remains paraelectric down to the lowest temperatures. In both SrTiO_3_ and KTaO_3_, the low temperature dielectric constant increases upon cooling but becomes temperature independent (<10 K for SrTiO_3_and KTaO_3_) due to quantum fluctuations. In both these systems, the quantum fluctuations suppress the ferroelectric instability and they enter a phase called quantum paraelectrics [1,2].

The substitution of ions of different valences and polarizabilities at both A and B sites in the parent perovskite compound results in the compositional disorder. This structural disorder gives rise to the formation of local dipolar impurities and defects that profoundly affect the static and dynamic properties of these materials [2]. The off centering of the A and/or B ion in the high polarizable perovskite host results in the development of net dipole moment in the unit cell. Each dipole polarizes the surrounding region, thus forming the local polar region called the polar nanoregion (PNR). The spatial correlation length *r_c_* determines the size of the PNRs. The flipping of polarization between the equivalent directions within the PNR gives rise to the relaxation behavior. In the dilute limit of substitution, *r_c_* is small, and each PNR behaves as an independent, noninteracting dipole with a single relaxation time. As substitution increases, so does *r_c_*, and the PNRs begin to interact with one another. The interaction between the PNRs results in complex relaxation behavior with a distribution of relaxation times. The average relaxation time then becomes strongly temperature dependent and exhibits divergent behavior with a decrease in temperature [2]. The dielectric constant (*ε_r_*) shows remarkable frequency dependence and broadening of the peak at *T_m_*, the temperature of the dielectric maximum. It is for this reason that the compositionally disordered ferroelectric materials are generally termed “relaxor ferroelectrics” or simply “relaxors.” In what follows, we discuss the important physical features exhibited by perovskite-type relaxor ferroelectric materials.

The relaxor behavior can be induced in a paraelectric host by the substitution of suitable ions at A and B sites, as in K_1−x_Li_x_TaO_3_, KTa_1−x_Nb_x_O_3_ and Sr_1−x_Ca_x_TiO_3_. Relaxorbehavior may also be intrinsic due to the compositional disorder at the B site as in Pb-based compounds. Pb-based relaxor ferroelectrics are Pb(Mg_1/3_Nb_2/3_)O_3_ (PMN), Pb(Mg_1/3_Ta_2/3_)O_3_ (PMT), Pb(Zn_1/3_Nb_2/3_)O_3_ (PZN), Pb(Sc_1/2_Nb_1/2_)O_3_ (PSN), Pb(Sc_1/2_Ta_1/2_)O_3_ (PST) and Pb(In_1/2_Nb_1/2_)O_3_ (PIN). Numerous studies have been carried out on the relaxation dynamics of PNRs in these disordered perovskite compounds. The temperature evolution of relaxation behavior of PNRs has been studied through dielectric, X-ray and neutron diffraction, thermal expansion, ultrasonic spectroscopy, inelastic neutron, and Raman and Brillouin scattering [3,4,5,6,7,8,9,10,11]. The characteristic feature that is related to the dynamical behavior of PNRs in the disordered perovskites is manifested in the temperature and the frequency dependence of the dielectric constant. The maximum dielectric constant ε_r_^max^ and its corresponding maximum temperature *T_m_*varies strongly with the frequency of the applied electric field. It is observed that ε_r_^max^ decreases while *T_m_* increases with the increase in frequency [6].

K_1−x_Li_x_TaO_3_ (KLT) exhibits relaxor behavior for the Li concentration up to 4%. Similar behavior is observed in KTa_1−x_Nb_x_O_3_ (KTN) for Nb substitution up to 2% [2]. In KLT, the large ionic radius difference between K (1.51 Å) and Li (0.92 Å) causes Li^+^ to be off-centered about 1.2 Å from ideal cubic position along the [100] direction. This results in the large dipole moment of Li^+^ ion and forms PNRs through the interaction with neighboring unit cells. Similarly, off-center displacement of Nb^5+^ ion in the highly polarizable KTaO_3_ host results in the formation of PNRs. Relaxor behavior has been observed for 1.5 and 3.5% substitution of Li in KLT through temperature and frequency-dependent dielectric studies [12]. Depending on the frequency of the applied field, both KLT and KTN show a broad dielectric maximum, with *T_m_* varying from 40 to 80 K. A strong deviation from Curie–Weiss behavior was observed in the temperature dependence of dielectric permittivity at the temperature T*, which is 20 K above *T_m_*. T* was identified as the temperature where PNRs enter into quasi-static regime. The symmetry-forbidden nonpolar Raman modes associated with PNRs appear for both KLT and KTN at T* [13,14]. The intensity of these nonpolar modes was found to increase with a decrease in temperature below T*. For both KLT and KTN, the average size of PNRs was estimated from the analysis of the Raman data [13,14]. The coupling between local polarization and strain in KTN was investigated through ultrasonic studies [9]. The elastic constants *C*_11_ and *C*_44_ exhibited a strong shift from the mean-field behavior at about T*. The deviation was attributed to the electrostrictive coupling between the local polarization and strain in PNRs. Recently, the ferroelectric phase transition in KLT crystals has been studied by broadband dielectric spectroscopy. Several different types of dipolar relaxations covering a broad temperature range have been observed [15]. 

Relaxor behavior has been observed in Sr_1−x_Ca_x_TiO_3_ (SCT-*x*) for small *x*. As the ionic radius of Ca^2+^ ion (1.12 Å) is considerably smaller than that of Sr^2+^ (1.26 Å), off-centering of Ca^2+^ ion at the Sr^2+^ site gives rise to the formation of PNRs. The evidence for the existence of Ca-induced PNRs was ascertained through dielectric, Raman scattering and nonlinear optical studies [16,17]. For small *x*, SCT show a glassy dipolar behavior and above the critical concentration of *x* = 0.0018, a long-range ferroelectric order was observed [18]. Based on dielectric and X-ray diffraction studies, a temperature–composition phase diagram was proposed for whole range of SCT solid solutions [19]. According to the phase diagram, SCT-*x* exhibits a ferroelastic-type phase transition from cubic to tetragonal structure at high temperature, and ferroelastic to ferroelectric relaxor transition accompanied by the change in structure from tetragonal to orthorhombic at low temperature [19]. At *x* = 0.04, the ferroelastic phase transition from cubic to tetragonal phase with the transition temperature of 210 K and the ferroelastic to ferroelectric relaxor transition at 30 K was observed through dielectric, X-ray and neutron diffraction studies [20]. Recent studies reveal the intriguing complexity of the physical behavior in oxygen deficient SCT. SCT-*x* becomes metal and shows superconducting behavior with the coexistence of both ferroelectric and superconducting phases for extremely small oxygen vacancies [21]. As in the case of SrTiO_3_, critical elastic anomalies are expected to occur in SCT-*x* at the ferroelastic phase transition temperature.

Pb-based perovskites Pb(B′_1/3_B″_2/3_)O_3_ and Pb(B′_1/2_B″_1/2_)O_3_ are a special group of relaxor ferroelectrics that exhibit complex structural and dynamical behaviors. Both types of relaxors have fixed chemical compositions and are intrinsically disordered at the B site. The antiparallel short-range displacement of Pb and B′/B″against oxygen octahedra along <111> direction leads to the formation of the PNRs [7]. In Pb-based relaxors, the nucleation of PNRs takes place at *T_B_*, the Burns temperature, which is much higher than *T_m_*. Non-Arrhenius behavior in the temperature dependence of relaxation time of PNRs was observed and the frequency dependence of the temperature of dielectric maximum *T_m_* was explained through the phenomenological Vogel–Fulcher relation [22]. The nucleation and further growth of PNRs below *T_B_* leads to the change in the thermal expansion, the deviation of Curie–Weiss law, specific heat, optical and acoustic phonon anomalies in Pb-based relaxors also [8,23,24,25]. In Pb-based relaxors, the temperature evolution of PNR dynamics was initially assumed to be characterized by two temperatures, namely *T_B_*, and *T_f_*, the freezing temperature. However, the detailed analysis of the Raman spectra of PMN, PZN and related relaxors revealed the existence of another intermediate percolation temperature, T* between *T_B_* and *T_m_* [10,26,27]. The observed anomalous variations in the frequency and intensity of Raman active phonon modes and the appearance of central peaks confirmed the existence of T*. T* is proposed as the onset temperature for the development of strong correlations between the initial quasi-dynamic PNRs. The temperature evolution of relaxation dynamics of PNRs consists of four different temperature stages: (i) a fully dynamic without any correlations above *T_B_*, (ii) quasi-dynamic phase between the temperature *T_B_* and T* with weak correlations, (iii) strongly correlated quasi-static phase between T* and *T_f_* and (iv) static below *T_f_* [26]. The existence of the intermediate percolation temperature T* was also confirmed through acoustic emission studies in PZN and related systems [28,29]. While the Burns temperature *T_B_* widely varies, T* was found to be almost same for all Pb-based relaxor ferroelectrics within the narrow temperature interval of 500 ± 30 K [30]. From the application point of view, the single crystals of relaxor-PbTiO_3_ (PT) solid solutions such as PMN-*x*PT, PZN-*x*PT and PIN-*x*PT with the composition at the morphotropic phase boundary (MPB) show giant piezoelectric effect, making them the ideal choice for ultrasonic transducers, actuators and micropositioners [31,32,33].

In contrast to Pb(B′_1/3_B″_2/3_)O_3_-type RFEs, the Pb(B′_1/2_B″_1/2_)O_3_ relaxor group exhibits varied ferroelectric behavior depending on the degree of B-site ordering [34,35]. The degree of B-site ordering can be altered by thermal treatment. The disordered (thermally quenched) PSN, PST and PIN show relaxor-type ferroelectric behavior [36,37,38]. While the ordered (thermally annealed) PSN and PST exhibit normal ferroelectric-like phase transition [36,37], the ordered PIN exhibit an antiferroelectric phase transition [38]. Both disordered PSN and PST undergo a spontaneous relaxor to the ferroelectric phase transition accompanied by the change in structure from cubic to rhombohedral at the temperature *T_c_* below *T_m_* [35,36,37]. The relaxor behavior becomes suppressed and the transition temperature decreases with increase in the degree of B-site ordering [37]. The nuclear magnetic resonance (NMR) studies revealed a substantial difference in the polarization dynamics between the disordered and the ordered regions of Pb(B′_1/2_B″_1/2_)O_3_ relaxor group [39,40]. NMR studies also revealed that the long-range polar order occurs only in fully B-site ordered region of the crystal [39,40].

In RFEs, a significant change in the dynamical behaviour of PNRs takes place in the quasi-static regime between T* and *T_f_*. This temperature range gives rise to the formation of static PNRs due to the strongly correlated shifts of A and/or B ions and associated quenched random dipolar fields.The local dipolar field results in the variation in the local strain field and both become coupled through quadratic electrostrictive effect. Brillouin light scattering spectroscopy is a valuable technique to study the coupling between the strain and polarization and relaxation dynamics of PNRs in relaxor ferroelectrics. The characteristic frequency range associated with the relaxation dynamics of PNRs is rightly located in the low frequency part of the Brillouin scattering, which is normally inaccessible through conventional Raman and thermal neutron scattering. The anomalous behavior of elastic constants in the quasi-static region in PMN and PMT was studied through Brillouin light scattering spectroscopy. Below T*, a broad central peak associated with the polarization fluctuations of PNRs was observed in PMN and PMT [41]. Both in PMN and PMT, the relaxation time of PNRs showed a characteristic slowing behavior with a marked anomaly below *T_f_*. The anomalous behavior of acoustic phonons and the central peak has been studied extensively in various Pb-based relaxor systems by Brillouin light scattering spectroscopy [42,43,44,45,46,47,48,49,50,51,52,53,54]. A significant softening of LA and TA phonon modes accompanied by a broad central peak was observed below T* in PZN-0.045PT and PZN-0.09PT [44]. In the Brillouin light scattering studies of PZN-*x*PT with *x* = 0.07, 0.1 and 0.12, a minimum was observed in the temperature derivative of the width of the LA phonon, dΓ/dT at T* [45]. Brillouin light scattering from acoustic phonons was investigated for a wide range of compositions in PMN-*x*PT solid solution [47]. As in the case of PZN-*x*PT, below T*, a significant softening of frequency of the LA phonon followed by increase in the width was observed. The activation energy of flipping of PNRs calculated using a modified superparaelectric model showed a dramatic increase across the composition of MPB. In 0.65PIN-0.35PT, anomalous behavior of the LA phonon mode at the cubic–tetragonal structural phase transition and two-component central peaks were studied using Brillouin light scattering spectroscopy [50]. The width of the LA phonon showed Landau–Khalatnikov-type behavior in the vicinity of the structural phase transition in a highly ordered PSN [52]. This review focuses on the Brillouin light scattering studies of dynamical aspects of PNRs in some selected RFEs carried out by the authors in the past. In the following, the results obtained in the Brillouin light scattering studies on RFEs such as PMT, PST, 0.65PIN-0.35PT and SCT-0.03 are reviewed in detail.

## 2. Brillouin Scattering

In Brillouin scattering, the incident photon undergoes inelastic scattering by the near-Brillouin zone-center acoustic phonons (*q* ≈ 0). The shift in the frequency of the scattered photon Δν_B_, known as the Brillouin shift is given by [53]:

(1) $$\Delta v_{B}=\pm \frac{2nV}{\lambda_{0}}sin\left(\frac{\theta }{2}\right)$$

where ± in the above Equation (1) represents the Stokes and anti-Stokes Brillouin scattering. *λ_0_, n, V* and *θ* correspond to the wavelength of the incident photon, refractive index, phase velocity of the acoustic phonon and the scattering angle between the incident and scattered photons, respectively. The intensity of the scattered light *I(q,ω)* at the given wave vector *q* and the frequency *ω* is related to the imaginary part of the complex dynamic susceptibility through the fluctuation–dissipation theorem, as given by [55]:

(2) $$I\left(q,\omega \right)=\frac{\hbar }{\pi }\left[n\left(\omega \right)+1\right]\;Im\chi^{*}\left(q,\omega \;\right)$$

where *n(ω)*, the Bose–Einstein thermal population factor, is given by:

(3) $$n\left(\omega \right)=\frac{1}{\exp\left(\frac{\hbar \omega }{k_{B}\;T}\right)-1}$$

where ħ is the Planck’s constant, *k_B_* the Boltzmann constant and T the temperature. For zone-center acoustic phonons, the dynamic susceptibility χ^*^(q,ω) is given by the Lorentzian response function:

(4) $$\chi^{*}\left(0,\omega \right)=\frac{f_{0}}{\omega^{2}-\omega_{0}^{2}-i\gamma_{0}\omega }$$

where *f_0_* is the oscillator strength, and *ω_o_* and *γ_o_* are the frequency and damping factor of the acoustic phonon. 

The high-contrast multi-pass Fabry–Perot interferometer (FPI) developed by Sandercock [56] is well suited to study Brillouin scattering in solids. A 3 + 3 pass tandem FPI provides adequate contrast and resolution to measure the frequency and the width of the acoustic phonons in solids in various scattering geometries. Since 3 + 3 pass FPI covers a large frequency range, 0.03–30 cm^−1^, not only the acoustic phonons but also the features of the central peak can be studied.

## 3. Materials and Methods

Single crystals of PMT, 0.65PIN-0.35PT and PST were grown by the flux method as described elsewhere [50,57,58]. Single crystals of SCT-0.03 were grown by flame fusion technique [59]. The degree of B-site ordering, *S* parameter, for PST was determined using powder X-ray diffraction. Brillouin light scattering measurements for all the crystals were performed using a high-contrast 3 + 3 pass Sandercock tandem Fabry–Perot Interferometer in backscattering geometry. Brillouin spectra were excited using a single longitudinal mode diode-pumped solid-state laser operating at 532 nm. Measurements were carried out in different free spectral ranges (FSR) in the temperature range 870–100 K using a Linkam (THMS 600) stage. The frequency and the width of acoustic phonons were deduced by fitting the experimental spectra with the Voigt function. During fitting, the Gaussian part of the Voigt function was kept constant to account for the instrumental broadening. The instrumental broadening was obtained by fitting the transmitted peak of the interferometer with the Gaussian function. The full width at half maximum of the Gaussian fit is the measure of the instrumental broadening. The central peak was fitted using a Lorentzian function with ω_o_ = 0.

## 4. Results and Discussion

### 4.1. Elastic Anomalies and Polarization Dynamics in PMT

**Figure 1:**
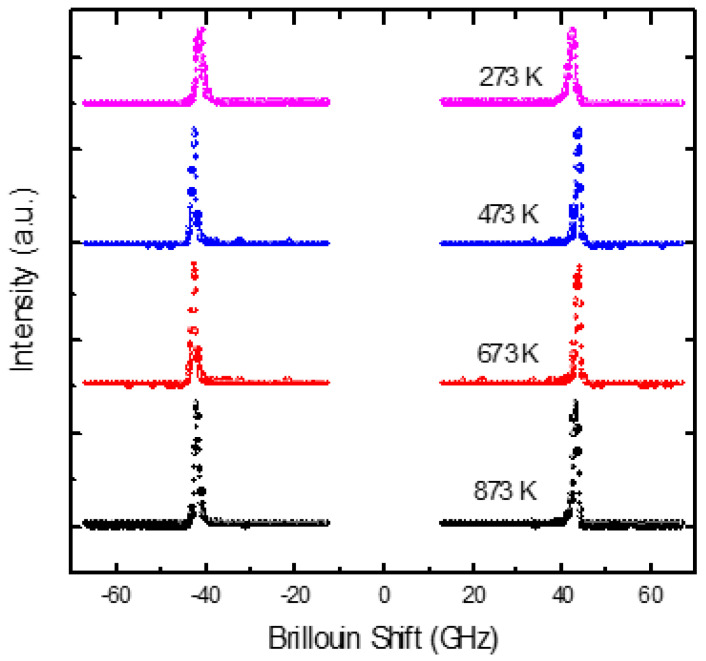
Brillouin spectra of PMT at different temperatures(Reprinted with kind permission from Ref. [41]. 2015, the *European Physical Journal* (EPJ)).

**Figure 2:**
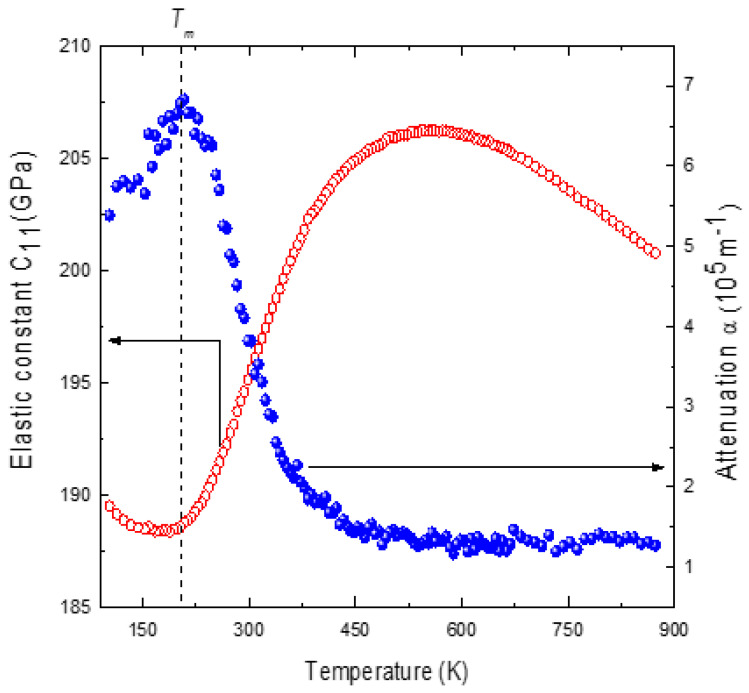
Temperature dependence of the elastic constant *C*_11_ (open circles) and attenuation α (closed circles) of PMT (Reprinted with kind permission from Ref. [41]. 2015, the *European Physical Journal* (EPJ)).

The Brillouin spectra of PMT measured in the FSR range of 75 GHz at different temperatures are shown in Figure 1. A single LA phonon mode with a frequency around 42 GHz can be seen. Since the macroscopic structure of PMT is cubic, the selection rule permits only the excitation of LA phonon mode in the backscattering geometry. The corresponding elastic constant *C*_11_ is given by the following expressions [60]:

(5) $$V_{11}=\frac{\lambda_{o}v_{LA}}{2n},\;C_{11}=\rho {\left(V_{11}\right)}^{2},\;\alpha =\frac{\pi \Gamma }{V_{11}}$$

where *ν_LA_*, *Γ*, *V*_11_ and *α* in Equation (5) are the frequency, width, velocity and attenuation of the LA phonon, and *ρ* and *n* are the density and the refractive index of PMT and *λ_o_* is the wavelength of the incident photon. Using the parameters of the LA phonon, *C*_11_ and *α* were calculated using the known values of *ρ* = 9.65 gm/cm^3^ and the refractive index *n* = 2.475 for PMT [60]. The temperature dependence of the refractive index was not considered in the calculation of *C*_11_, as it does not significantly affect its magnitude. Figure 2 shows the temperature dependence of *C*_11_ and *α* for PMT.

The elastic constant *C*_11_ shows a broad minimum and *α* shows a broad maximum in the region of the dielectric anomaly at *T_m_*. Our data are in good agreement with those of the earlier studies on the elastic anomalies observed in PMT [60]. The minima of *C*_11_ agree well with the corresponding dielectric anomaly of the PMT [42]. In the present review, a mean field model that was previously used to study the coupling between the polarization and the strain in KTN is employed [9]. The temperature dependence of *C*_11_ according to this model is given by the following expression:

(6) $$C_{11}=A_{1}-A_{2}T-A_{3}{\left(T-T_{o}\right)}^{-p}$$

**Figure 3:**
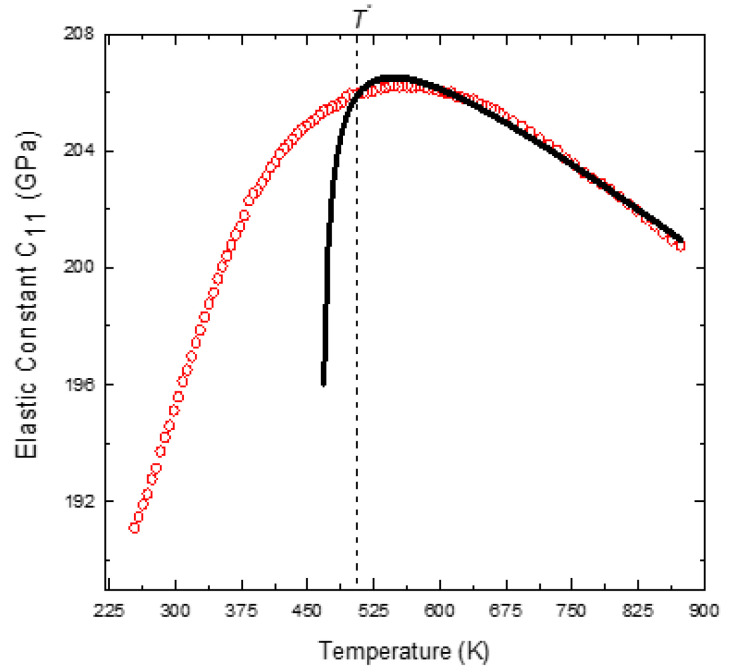
Fit of the experimental *C*_11_ (open circles) with Equation (6) (solid line) of PMT (Reprinted with kind permission from Ref. [41]. 2015, the *European Physical Journal* (EPJ)).

The first two terms of Equation (6) describe the normal anharmonic process, and the third term accounts for the critical softening of *C*_11_ due to the critical fluctuations. For three-dimensional (3D) fluctuations, the critical exponent *p* = 0.5, for 2D and 1D fluctuations; it is 1 and 1.5 [61]. Since PNRs begin to appear around *T_B_*, the temperature dependence of *C*_11_ was fitted with this equation for temperatures higher than *T_m_*. As the structure of PMT is cubic, it is reasonable to assume *p* to be 0.5. *C*_11_was therefore fitted with the critical exponent *p* fixed at 0.5 and all the other parameters in Equation (6) are allowed to vary freely [9]. The parameters obtained from the fitting are: A_1_ = 223.45 GPa, A_2_ = 0.024 GPa/m, A_3_ = 36.5 GPa and *T_o_* = 463 K. Figure 3 shows the result of the fitting for PMT.

The experimental *C*_11_ shows good agreement with the mean-field model in the temperature above *T_o_* + 40 K. *C*_11_ shows a marked deviation from the mean-field model below this temperature and exhibits reduced softening. The attenuation α also begins to rise precisely at the temperature where *C*_11_ deviates from the mean field model. The temperature at which *C*_11_ begins to show reduced softening can be identified with the characteristic percolation temperature T* [26]. T* obtained for PMT is 503 K, which is well in agreement with that of the X-ray diffraction data [30]. The temperature dependence of *C*_11_ between *T_B_* and T* is determined by the third term of Equation (6), which accounts for the quasi-dynamic fluctuations inpolarization.

The reduced softening of *C*_11_ and increased α below T^*^ indicates the appearance of excess strain in the crystal. This additional strain Δ*S* arises from the electrostrictive coupling between the polarization and strain and is expressed as $\Delta S=\left(\gamma_{11}+2\gamma_{12}\right)\langle P^{2}\rangle$, where γ*_11_* and γ*_12_* are the electrostriction coefficients [2]. Equation (6) does not account for the excess contribution from the electrostriction γ to the elastic constant *C*_11_. The reduced softening of *C*_11_ below T* can be qualitatively explained using Kubo’s formalism [62]. According to this formalism, the polarization fluctuations *δP* contain two components with very distinct dynamics:

(7) $$\delta P=\delta P_{d}+\delta P_{s}$$

Here, *δP_d_* is the quasi-dynamic part, i.e., the third term of Equation (6), and *δP_s_* corresponds to much slower fluctuations (quasi-static) with the development of long-range correlations between the PNRs. The quasi-static part *δP_s_* is expressed in terms of the Edwards–Anderson order parameter $q^{EA}=V^{-1}\int \langle \delta P_{s}\rangle {}^{2}dV$, where *V* is the volume over which long-range correlations exist [62,63]. The change in the elastic constant Δ*C* in the quasi-static region can be written as [63]:

(8) $$\Delta C_{s}=-4{\left(R_{11}+R_{12}\right)}^{2}q^{EA}\chi^{\prime }\chi^{\prime \prime }$$

where *χ*′ and *χ*″ are the real and imaginary parts of the low-frequency dielectric susceptibility. In the disordered Rb_1−x_(NH_4_)_x_H_2_PO_4_, the quasi-static term of Equation (8) makes a dominant contribution to the anomalous region of the frequency and width of LA phonon mode [62,63]. A detailed study of the contribution ofthe quasi-static term Δ*C_s_* on the temperature dependence of *C*_11_ for Pb-based relaxors is yet to be carried out. However, a qualitative idea of the temperature dependence of Δ*C_s_* can be obtained from the study of *q^EA^*. The direct determination of *q^EA^* by ^93^Nb NMR for PMN shows a strong increase with a decrease in temperature below T* [64]. The deviation of the elastic constant *C*_11_ from the mean field behavior below T* therefore evidently arises from the additional term Δ*C_s_* of the quasi-static polarization within PNRs. In fact, in PMN, the reduced softening of *C*_11_ almost coincides with the rise inthe local polarization *P_d_* below T* [65]. Therefore, in PMT, the reduced softening of *C*_11_ and the increased attenuation in the quasi-static region are evidently associated with the appearance of additional strain due to the strong coupling between the polarization and the strain.

**Figure 4:**
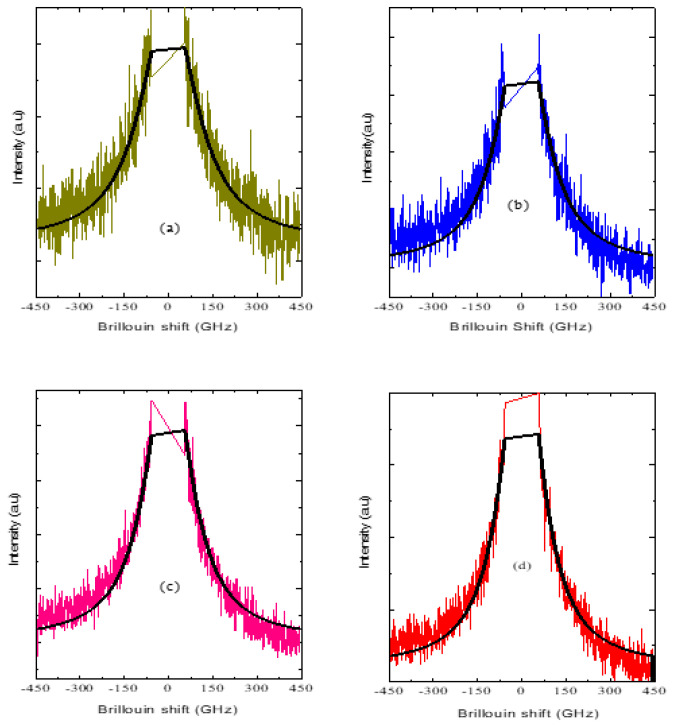
(**a**–**d**)Typical broadband Brillouin spectra of PMT at (**a**) 293 K, (**b**) 273 K, (**c**) 253 K and (**d**) 223 K.The solid black line is the Lorentzian fit of the experimental data.

**Figure 5:**
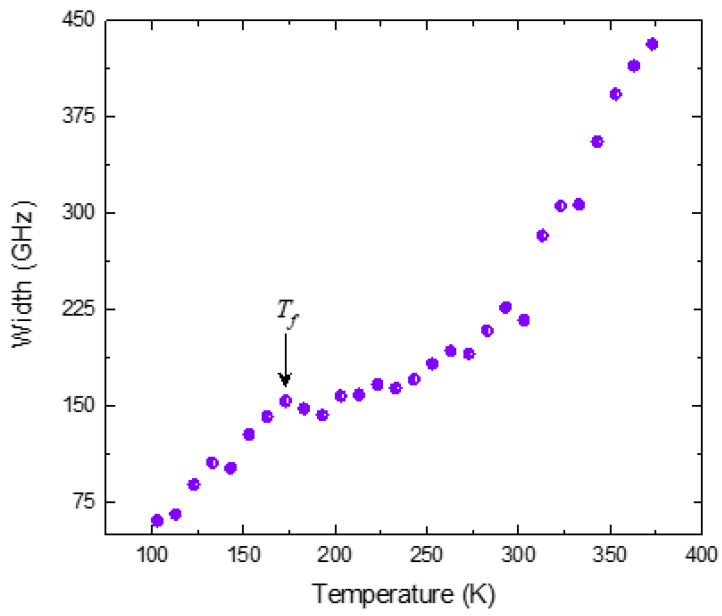
Temperature variation inthe width of the central peak of PMT (Reprinted with kind permission from Ref. [41]. 2015, the *European Physical Journal* (EPJ)).

In PMT, a broad central peak with marked temperature dependence appears below T*. The typical central peak spectra measured in the FSR range of 500 GHz at different temperatures are displayed in Figure 4a–d. The intensity of the central peak can be described as a Debye-type relaxation according to the following equation [66,67]:

(9) $$I\left(\omega \right)\propto \frac{\omega \tau }{\left(1+\omega^{2}\tau^{2}\right)}$$

where *τ* is the relaxation time.In the strong damping limit, (γ_o_>>ω_o_), the Lorentzian function given by Equation (4) reduces to the Debye-type function with ω_o_ = 0 [55]. The width of the central peak obtained from the fit of the Lorentzian function is then proportional to 1/*τ*. The width of the central peak obtained from the fit is shown in Figure 5. With the decrease in temperature below T*, the width decreases with a change in slope between *T_m_* and 160 K. An anomalous decrease in the width of the central peak can be observed below 160 K. This temperature corresponds to the freezing temperature *T_f_* for PMT [68]. The relaxation time of the central peak corresponds to the short relaxation time *τ_s_* obtained from the broadband dielectric measurements [68].

**Figure 6:**
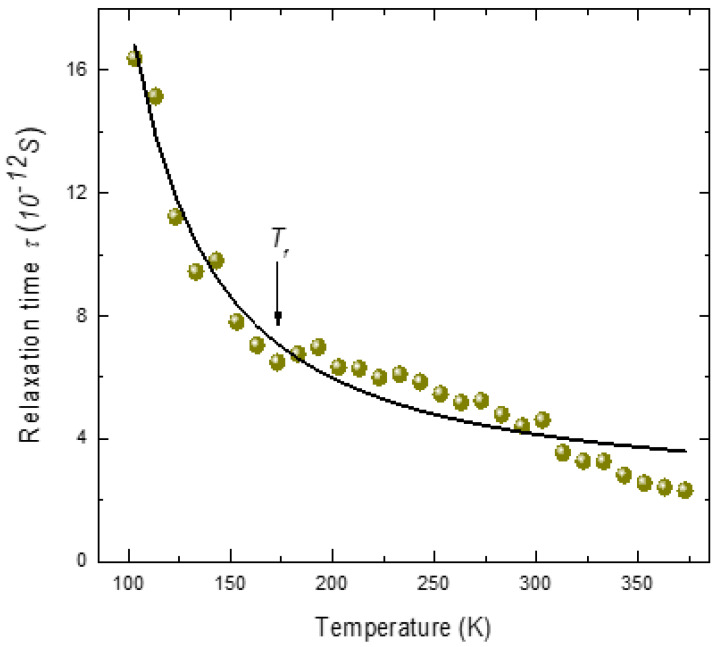
Fit of the Arrhenius law (solid line) with the relaxation time *τ* (solid sphere) (Reprinted with kind permission from Ref. [41]. 2015, the *European Physical Journal* (EPJ)).

To obtain a qualitative understanding of the relaxation behavior, the temperature dependence of relaxation time was analyzed using the simple Arrhenius law *τ = τ_o_*exp[*E_a_*/kT], as shown in Figure 6. The parameters obtained from the fit are *τ_o_* = 2.0 ± 0.14 × 10^−12^*S*, *E_a_* = 220 ± 9 K for PMT. Though the fitting parameters are reasonable, the Arrhenius behavior does not agree well with the experimental data. The observed non-Arrhenius behavior suggests that the relaxation dynamics are not governed by dipolar fluctuations alone. Broadband dielectric studies in Pb-based relaxor systems suggest that the relaxation dynamics of PNRs are not governed by a single relaxation mechanism [68,69,70]. According to these studies, the dominant mechanism of relaxation between T* and *T_f_* is the dipole flipping, while that below *T_f_* is the volume fluctuations (breathing) of PNRs [68,69,70]. The strong increase in the relaxation time below *T_f_* suggests that the relaxation mechanism above *T_f_* is different from that below *T_f_*. Therefore, the observed critical slowing of the relaxation time of the central peak between T* and *T_f_* can be attributed to the dipole flipping and below *T_f_* to the volume fluctuations of PNRs.

This interpretation seems to be quite consistent with the existence of different stages of relaxation dynamics of PNRs as revealed by Raman scattering studies [26]. The increase in correlations between PNRs in the quasi-static regime between T* and *T_f_* increase the relaxation time. In the temperature range below *T_f_*, due to the freezing, the dipolar flipping in PNRs is no longer possible. Therefore, the observed anomalous increase in the relaxation time below *T_f_* could be due to the volume fluctuations of PNRs.

### 4.2. Anomalous Behavior of Longitudinal Acoustic Phonons in PST

**Figure 7:**
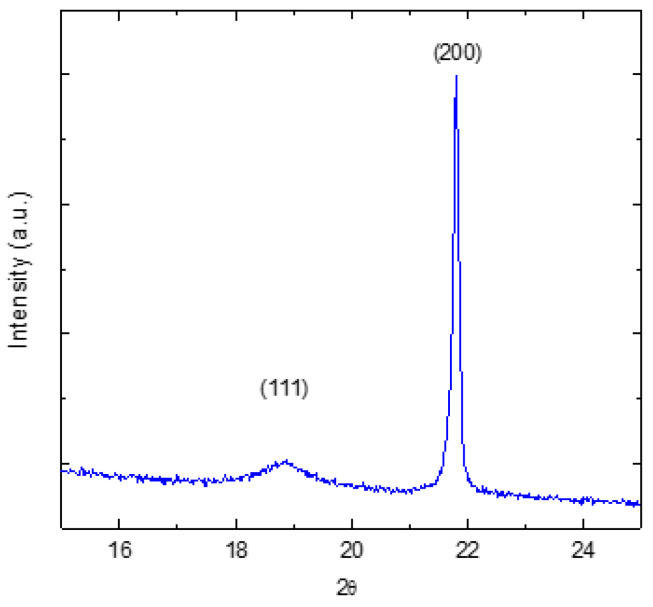
Powder X-ray diffraction of PST.

Figure 7 shows the powder X-ray diffraction pattern of PST. The broad superlattice reflection due to the B-site ordering is observed at 2θ = 19^o^.

**Figure 8:**
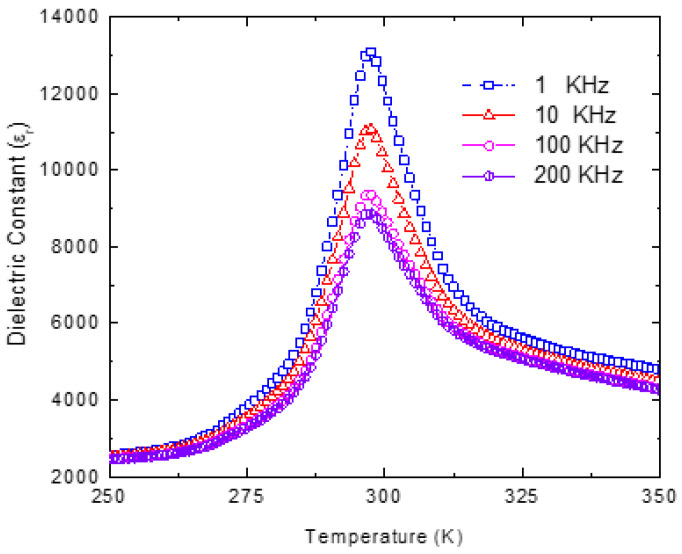
Low frequency dielectric dispersion of PST.

The degree of B-site ordering $S={\left[{\left(\frac{I_{111}}{I_{200}}\right)}^{1/2}\right]}_{\exp}$/${\left[{\left(\frac{I_{111}}{I_{200}}\right)}^{1/2}\right]}_{theo}$ was calculated from the ratio of the integrated intensities of the superstructure (111) and the adjacent fundamental (200) peaks [70]. Here,${\left(\frac{I_{111}}{I_{200}}\right)}_{exp}$ is the experimental intensity ratio and ${\left(\frac{I_{111}}{I_{200}}\right)}_{theo}$ is the theoretical intensity ratio for the completely ordered PST. The theoretical intensity ratio *I*_111_/*I*_200_ for the completely ordered PST is estimated to be 1.33 [71]. The S parameter for the as-grown PST single crystals is found to be *S* = 0.55. This indicates that the as-grown PST single crystal is partially disordered. Figure 8 shows the low-frequency dielectric dispersion of partially ordered PST. Since it is known that an increase in B-site ordering suppresses the relaxor behavior, the partial ordering in PST results in the weak relaxor-like behavior with *T_m_* = 297 K.

**Figure 9:**
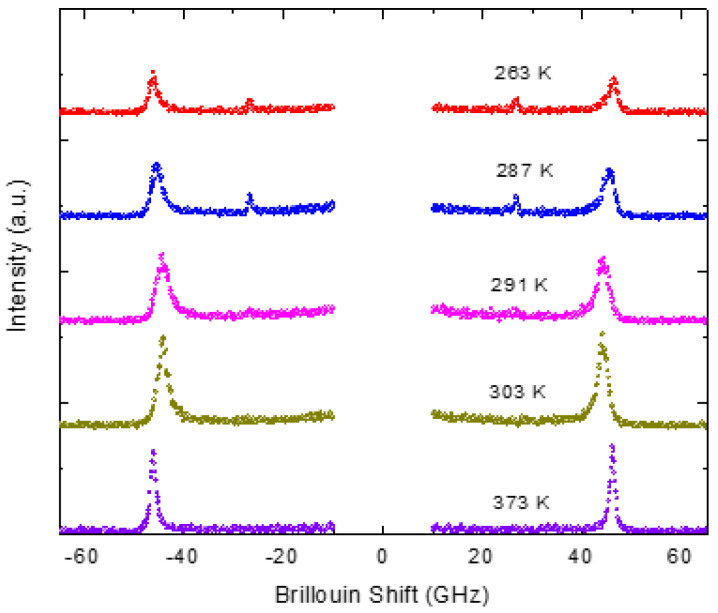
Brillouin spectra of PST measured in the FSR range of 75 GHz.

The typical Brillouin spectra of PST measured in the FSR range of 75 GHz at different temperatures are displayed in the Figure 9. In the cubic phase above 295 K, only the LA phonon mode could be observed at the backscattering geometry. Since PST exhibits a spontaneous phase transition from cubic to rhombohedral structure [72], a transverse acoustic (TA) phonon mode at 27 GHz, allowed by symmetry, appears below 295 K in the rhombohedral phase. However, the intensity of the TA phonon was too weak to obtain a reliable value for the frequency and width. Hence, the temperature dependence of TA mode is not discussed further.

**Figure 10:**
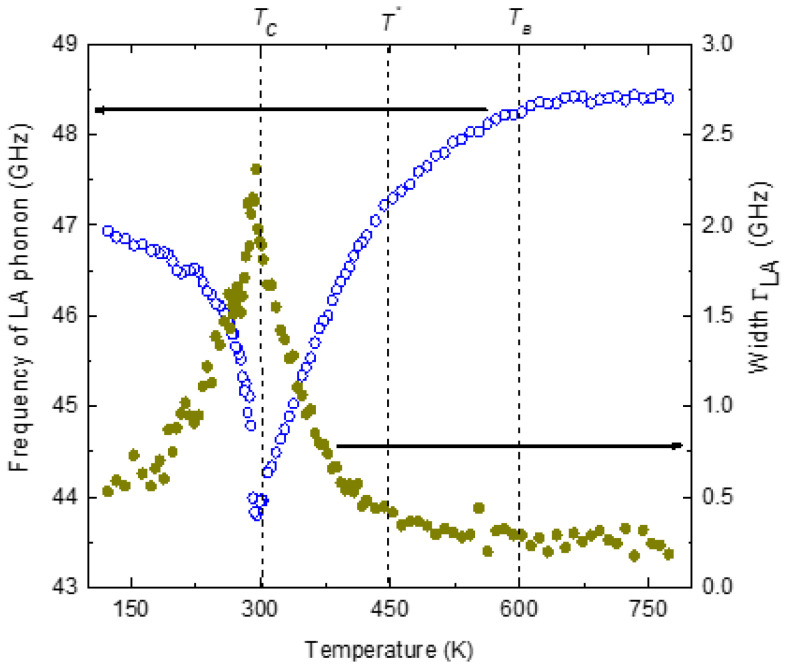
Temperature variation in frequency (open circles) and the width(closed circles) of the LA phonon of PST.

Figure 10 displays the temperature variation in frequency ω_LA_ and the width *Γ*_LA_ of the LA phonon. Above 600 K, the frequency and the width of the LA phonon are almost independent of temperature. The LA phonon mode begins to deviate from the normal high-temperature behavior below 600 K and exhibits marked softening with a sharp minimum at *T_c_* = 295 K, the transition temperature. The width of the phonon mode begins to show an anomalous increase at 450 K with a sharp maximum at *T_c_*. 

Interaction between the acoustic phonon and polarization affects the static and dynamic parts of the complex elastic constant *C*. Depending on the form of the interaction energy, the interaction can be classified as either linear or nonlinear. For bilinear coupling, the interaction energy *F_c_* = *βSP*, where *S* is the strain, *P* is the polarization and *β* is the piezoelectric coefficient. *F_c_ = γSP^2^* for electrostrictive coupling, where *γ* is the electrostrictive coefficient. The coupling between the strain and polarization affects both the static and dynamic parts of the complex elastic constant *C*, as given by [73,74,75,76]:

C^*^(ω) = *C_∞_* − Δ*C* (10)

In Equation (10), *C_∞_* is the elastic constant far from the transition temperature and Δ*C* is the change in elastic constant due to the coupling between strain and polarization. For bilinear coupling, the elastic constant softens as */T − T_c_/*^−1^ on both sides of the transition. For electrostrictive coupling, the elastic constant abruptly decreases to a lower value at *T_c_* [73,76]. Brillouin scattering measures the dynamic part of *C* according to Equation (11):

*C(ω)* = *ρV*^2^*(ω)* (11)

where *ρ* is the bulk density of the crystal and *V* is the corresponding velocity of the acoustic phonon. Assuming a Debye-type relaxation, the dynamic behavior of *C* is given by the following form [74,75]:

(12) $$\;C^{*}\left(\omega \right)=C_{\infty }-\frac{\Delta C}{1-i\omega \tau }$$

where *τ* is the relaxation time. The frequency of the acoustic phonon is related to the real part of the elastic constant, while the width is related to the imaginary part of the above equation, as given by:

(13) $$Re\;C\left(\omega \right)=C_{\infty }-\frac{\Delta C}{1+\omega^{2}\tau^{2}}$$

(14) $$\Gamma =\frac{q}{2\pi \rho V}\left(Im\;C\right)=\Gamma_{\infty }+\frac{\Delta C\tau q^{2}}{2\pi \rho \left(1+\omega^{2}\tau^{2}\right)}$$

The relaxation mechanism is also known as Landau–Khalatnikov (LK) mechanism. The line shape of the width of the LA phonon, as shown in Figure 10, is markedly asymmetric about *T_c_*, as expected from the LK mechanism.

**Figure 11:**
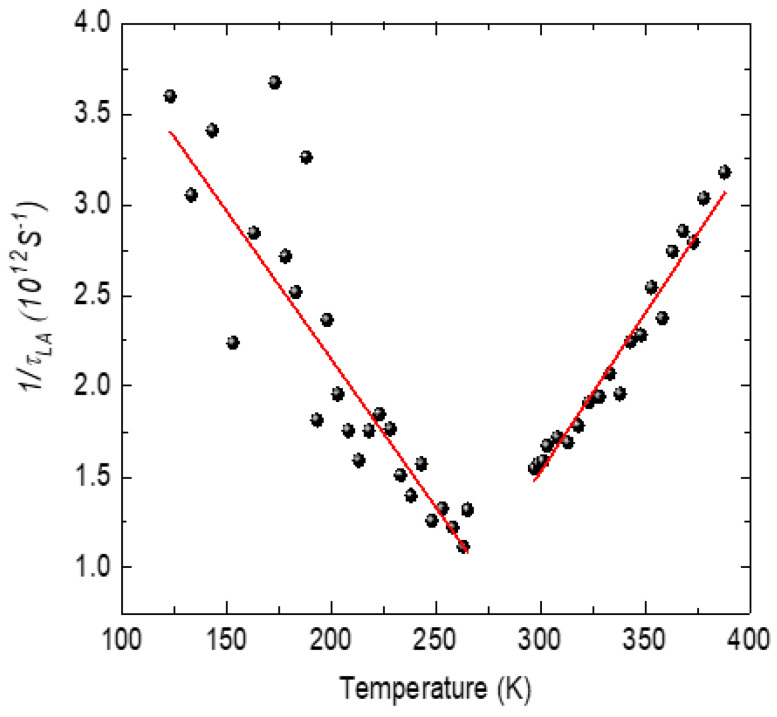
Plot showing 1*/τ_LA_* as a function temperature for the LA phonon of PST. Points are the experimental data and the solid line is the fit of Equation (16).

Using Equations (5), (13) and (14), the relaxation time can be expressed as:

(15) $$\frac{1}{2\pi \tau }=\frac{\upsilon_{\infty }{}^{2}-\upsilon^{2}(T)}{\Gamma (T)-\Gamma_{\infty }}$$

where υ*(Γ)* is the frequency (width) of the acoustic phonon at a given temperature and $\upsilon_{\infty }$*(Γ_∞_)* is the corresponding value of the acoustic phonon far above the transition temperature. In the relaxor-like system Rb_1−x_(NH_4_)_x_H_2_PO_4_with x= 0.35, the temperature dependence of the width of the LA phonon in the quasi-static region was shown to be equivalent toan LK-type mechanism [62]. Similarly, in the relaxor phase of the PST, the anomalous increase in the width of the LA phonon below T*is evidently connected with the development of the quasi-static part *δP_s_*. Therefore, a linear coupling between the polarization and strain should exist in the quasi-static region below T* [62]. Moreover, according to Young and Scott, Equation (12) applies to any form of coupling, such as bilinear or electrostrictive coupling and the coupling between the acoustic phonon mode and other excitations, which are altogether not connected with the order parameter [76]. This relaxation mechanism was used to describe the relaxation behavior of the acoustic phonon mode across commensurate–incommensurate phase transition in Ba_2_NaNb_5_O_15_ where the exact form of the coupling was not known [76,77]. Based on the above studies in Rb_1−x_(NH_4_)_x_H_2_PO_4_ and Ba_2_NaNb_5_O_15_, we calculated the relaxation time both in the ferroelectric phase below *T_c_* and in the quasi-static region below T* (in the temperature range between T* and *T_c_*) using the LK relaxation mechanism. Figure 11 shows the temperature variation inthe relaxation time *τ_LA_*of the LA phonon.

The relaxation time exhibits a critical slowing on approaching *T_c_* from the ferroelectric and the relaxor phase. As shown in Figure 11, the temperature variation inthe relaxation rate exhibits approximately linear behavior where 1/*τ_LA_* satisfies the following relation:

(16) $$\frac{1}{\tau_{LA}}=\frac{1}{\tau_{o}}\frac{\left|T-T_{c}\right|}{T_{c}}$$

The solid line is the linear fit to the experimental data. Best fit of the experimental data with Equation (16) yields *τ_o_* = 0.22 ps in the ferroelectric phase and 0.19 ps in the relaxor phase. The relaxation time *τ_o_* for PST is of a similar order of magnitude as observed for typical order–disorder ferroelectrics such as potassium dihydrogen phosphate KH_2_PO_4_ (KDP) and triglycine sulfate (NH_2_CH_2_COOH)_3_H_2_SO_4_ (TGS); *τ_o_* is 0.13 ps for KDP [78] and 0.10 ps for TGS [79]. Since the structural phase transition in PST does not involve the condensation of soft mode, the transition can be of the order–disorder-type. The local structure of PNRs in Pb-based relaxors is known to be rhombohedral [7] and sufficiently strong interaction between the quasi-static PNRs in the temperature range below T* may eventually lead to the formation of long-range ferroelectric order with the macroscopic rhombohedral structure below *T_c_*.

### 4.3. Brillouin Spectroscopy of Cubic–Tetragonal Structural Phase Transition in a 0.65PIN-0.35PT Single Crystal

PIN-PT solid solution, like PMN-PT and PZN-PT, shows the sequence of structural transition with temperature and PT content and has MPB for x = 0.37 [80]. PIN-PT composition near MPB shows a structural phase transition from cubic to tetragonal (*T_ct_*) and tetragonal to rhombohedral (*T_tr_*) phases [43,81]. While the dynamical behavior of PNRs has been widely studied in the PMN-PT and PZN-PT systems, no such studies have been carried out for PIN-PT solid solutions. In this review, the Brillouin scattering studies of the relaxation dynamics of PNRs in 0.65PIN-0.35PT single crystals are discussed.

**Figure 12:**
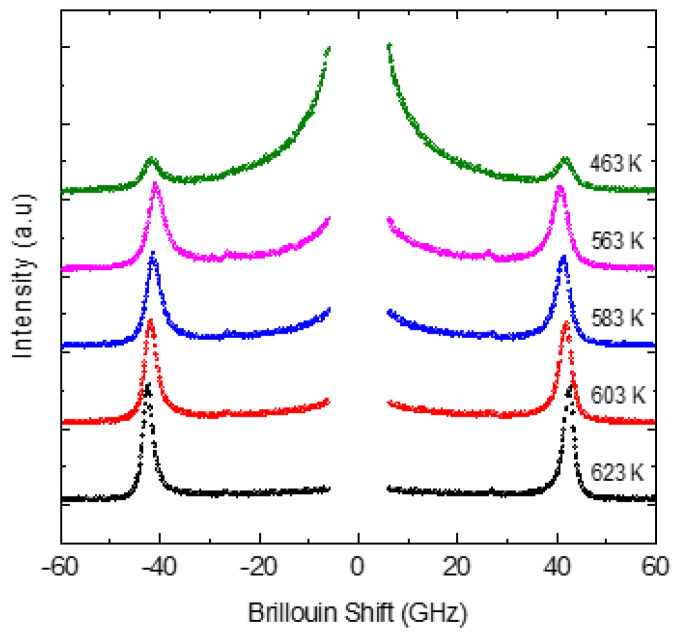
Brillouin spectra of 0.65PIN-0.35PT at various temperatures.

**Figure 13:**
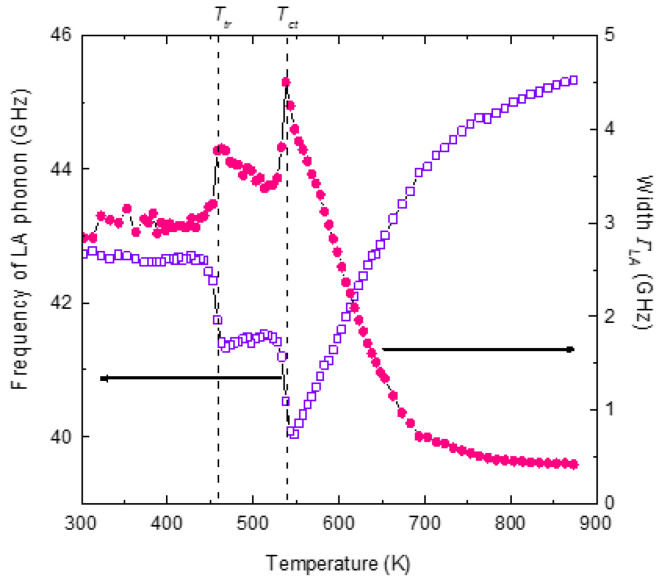
Temperature variation in the frequency and the width of the 0.65PIN-0.35PT LA phonon.

The Brillouin spectra measured at various temperatures in the FSR range of 75 GHz are shown in Figure 12. The Brillouin doublet of the LA phonon can be seen clearly. A faint and broad TA phonon mode can also be observed in the cubic phase. This could be due to the presence of tetragonal-structure PNRs. The TA mode, however, could not be followed for all the temperatures and hence is not discussed further. Figure 13 shows the frequency and the width *Γ*_LA_of the LA phonon as a function of temperature. Above 700 K, the minor temperature dependence in the frequency and the width of the LA phonon is attributed to the normal anharmonic process. Below 700 K, the frequency of the LA phonon shows substantial softening, followed by a strong increase in its width on approaching cubic to tetragonal phase transition temperature *T_ct_* = 540 K. Also clear are the LA anomalies that can be seen at the tetragonal–rhombohedral transition temperature *T_tr_* = 460 K. 

**Figure 14:**
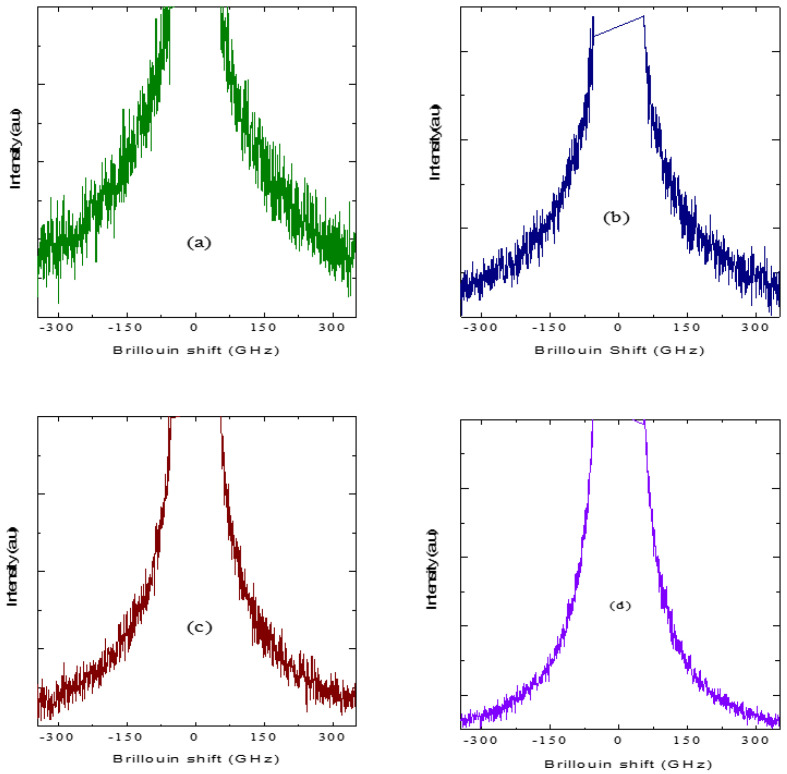
(**a**–**d**)Broad central peak of 0.65PIN-0.35PT at (**a**) 633 K, (**b**) 593 K, (**c**) 573 K and (**d**) 463 K.

**Figure 15:**
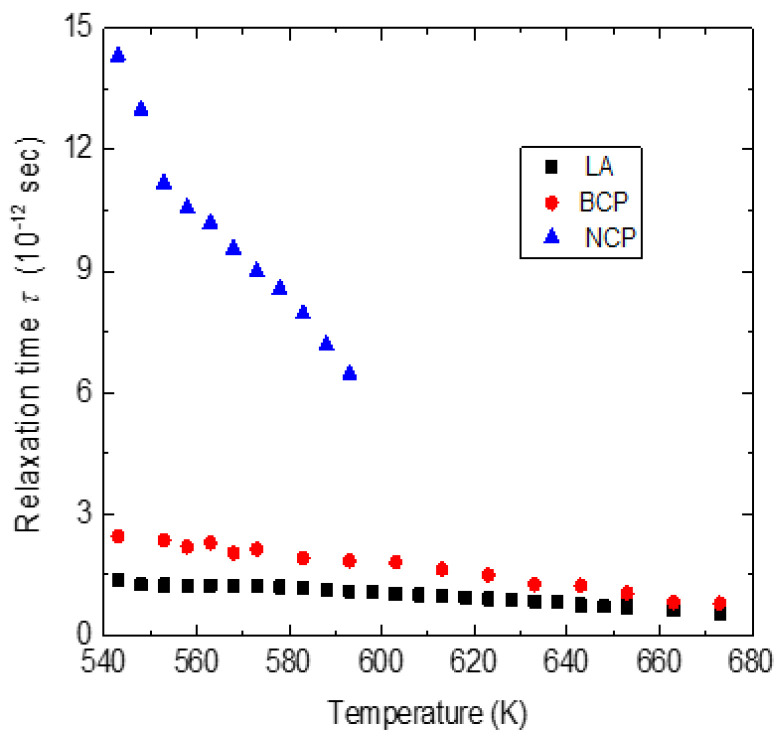
Temperature dependence of relaxation time of LA (solid squares), broad (solid circles) and narrow (solid triangles) central peaks of 0.65PIN-0.35PT.

The observed anomalous behavior in the frequency and the width of the LA phonon below 700 K clearly suggests that *T_B_*~700 K. As discussed in the Introduction, the strong electrostrictive coupling between the local polarization and strain in PNRs leads to the anomalous behavior of the LA phonon below *T_B_*. As can be seen from Figure 12, a narrow CP (NCP) begins to appear at about 620 K and its intensity grows further substantially upon cooling. Figure 14a–d show the Brillouin spectra measured in the FSR range of 400 GHz. A broad CP begins (BCP) to appear below *T_B_* and its intensity increases with a further decrease in temperature. The width of both NCP and BCP decreases upon cooling toward *T_ct_*, reflecting the growth of the PNRs and the corresponding slowing of the relaxation time. The relaxation time of the LA phonon was calculated using Equation (15). We use the highest temperature value of the frequency and width of the LA mode for *ν_∞_* and *Γ_∞_*. In Figure 15, the calculated value of the relaxation time from the above expression is shown along with the relaxation times obtained for narrow and broad central peaks above *T_ct_*.

The relaxation time for the LA phonon in the ferroelectric phase was not calculated due to the lack of sufficient data points. Below 600 K, the relaxation time of the local strain, *τ_LA_* qualitatively agrees with the polarization fluctuations (as determined by BCP) of PNRs. Though the broad central peak appears below *T_B_*, it effectively becomes coupled with the LA phonon only from 600 K. This result implies that the relaxation process of PNRs below 600 K is determined by the polarization fluctuations coupled with the local strain fluctuations. A similar result was obtained for 0.71PNN-0.29PT [82]. In this solid solution system, broad and narrow central peaks were observed around T* = 500 K. The relaxation time of the LA phonon agrees well with that of the broad central peak. Considering the similarity of relaxation dynamics with 0.71PNN-0.29PT, we suggest that for 0.65PIN-0.35PT, the temperature 600 K at which the narrow central peak appears can be taken as T*. At this point, we would like to remark on the two-component (narrow and broad) central peaks observed in 0.65PIN-0.35PT. Two-component central peaks appear to be the common feature in all the PT-substituted relaxor solid solutions [44,74,82]. We suggest the possible origin of two-component central peaks in the relaxor–PT solid solution. Owing to the high polarizability of the Ti^4+^ ion, the substitution of PT may lead to the formation of PNRs with a tetragonal structure in addition to the already existing PNRs with rhombohedral structure. With decrease in temperature, the size of tetragonal PNRs may grow faster and eventually become larger than the size of the rhombohedral one. In this case, the activation energy required for the flipping of dipoles along equivalent directions in tetragonal PNRs becomes higher. The higher activation energy results in a longer relaxation time for tetragonal PNRs. Therefore, we attribute the narrow component of the central peak to the relaxation of tetragonal PNRs and the broad component to the rhombohedral PNRs. It may be noted that in both PST and PSN, increased correlations between rhombohedral PNRs anddecrease in temperature results in long-range ferroelectric order with the macroscopic rhombohedral structure below *T*_c_. In both PSN and PST, only a broad central peak corresponding to rhombohedral PNRs was observed below T*[46,51]. Therefore, we suggest that in 0.65PIN-0.35PT, the phase transition from cubic relaxor phase to the tetragonal ferroelectric phase could be due to the nucleation and growth of tetragonal PNRs, whose relaxation dynamics are different from that of the rhombohedral PNRs.

### 4.4. Brillouin Light Scattering Studies of Acoustic Phonon Anomalies in SCT-0.03 Quantum Ferroelectrics

**Figure 16:**
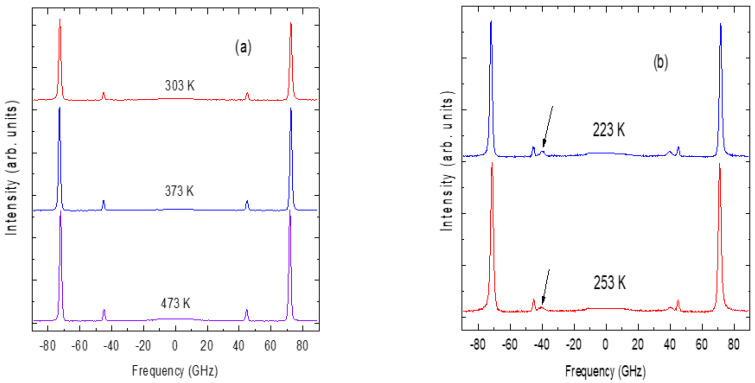
Brillouin spectra of SCT-0.03 measured with FSR of 100 GHz at (**a**) 473, 373 and 303 K and (**b**) 253 and 223 K. (Figure 16a. Reprinted with kind permission from Ref. [83]. Copyright (2020) The Japan Society of Applied Physics).

**Figure 17:**
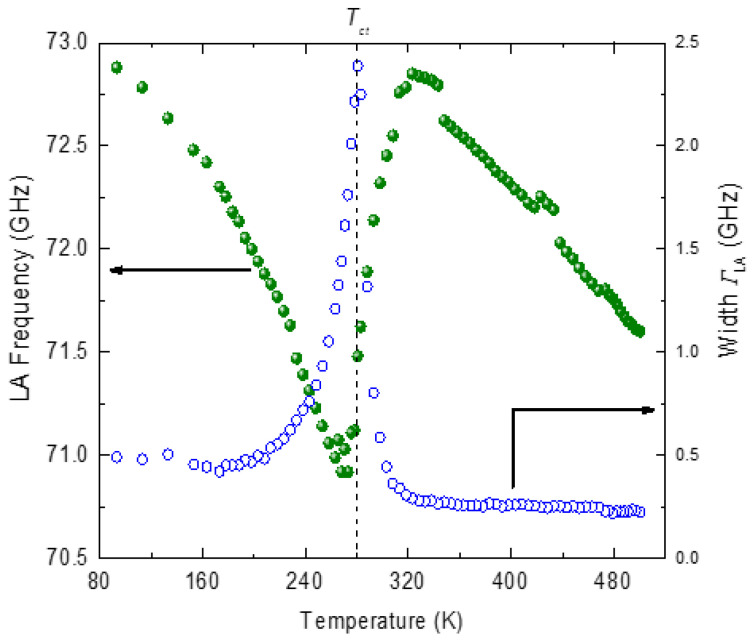
Temperature variation inthe frequency and the width of the SCT-0.03LA phonon. (Reprinted with kind permission from Ref. [83]. Copyright (2020) The Japan Society of Applied Physics).

The Brillouin spectra of SCT-0.03 in some temperature ranges are displayed in Figure 16a,b. A longitudinal acoustic (LA) phonon peak around 75 GHz and a transverse acoustic (TA) mode at 45 GHz can be seen. As shown in Figure 16b, the TA mode splits into two components (TA1 and TA2) below 280 K (indicated by the arrow mark). The temperature variation in the frequency and the width of the LA phonon is shown in Figure 17. In the temperature range 500–323 K, a linear increase in the frequency of the LA phonon with the near constancy of its width is ascribed to the normal anharmonic process. Below 323 K, the LA phonon shows anomalous behavior with a sharp minimum in the frequency followed by a sharp maximum in width at the ferroelastic phase transition temperature *T_ct_* = 280 K [83]. Similar elastic anomalies are observed in nondoped SrTiO_3_ crystal by picosecond ultrasound spectroscopy [84]. The remarkable increase in *T_ct_* by Ca doping can be caused by the smaller ionic radius of Ca ions, which induces the antiferro distortive rigid rotation of the oxygen octahedral [85]. A similar increase in *T_ct_* was also reported in La-doped SrTiO_3_ [86].

**Figure 18:**
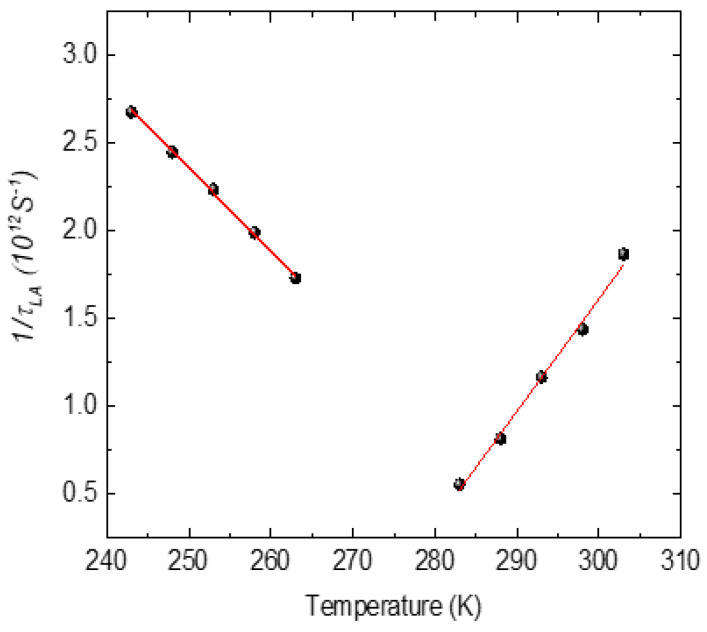
Plot showing 1*/τ_LA_* as a function temperature for the LA phonon of SCT-0.03. The solid line shows the fit of the experimental data points.

The width of the LA phonon is markedly asymmetric in the vicinity of the transition temperature as expected from the LK mechanism. In the X-ray and the neutron diffraction studies of SCT-0.04, the cubic to tetragonal phase transition with the transition temperature of 210 K was reported [20]. The phase transition in SCT-0.04 was observed to be a ferroelastic-type as it was not associated with any dielectric anomalies. Therefore, the observed anomalous behavior of the LA phonon at 280 K in SCT-0.03 in the present study is attributed to the ferroelastic-type structural phase transition from cubic to tetragonal phase. The higher transition temperature in SCT-0.03 could be due to the lower Ca content. The observed anomalies of the LA phonon in the phase transition region are caused by the interaction between the phonon mode and the order parameter. It can be seen from Figure 17 that the order parameter interaction with the LA phonon does not agree with the bilinear coupling that requires */T −T_ct_/*^−1^-type temperature dependence on either side of the phase transition. Slonczewski and Thomas reported that the ferroelastic phase transition at 110 K in SrTiO_3_ can be described by coupling between the strain *S* and the order parameter *Q* in the form *F_c_ = δSQ^2^*, where the order parameter *Q* is the soft mode coordinate and *δ* is the coupling coefficient [87]. The ferroelastic transition in SrTiO_3_ could be well described by Landau theory, taking into account the interaction between the strain and the soft optical phonon mode.We suggest that in SCT also, the observed acoustic anomaly could be due to the coupling between the LA phonon and the soft mode. The temperature dependence of the relaxation time *τ_LA_* of the LA phonon, calculated using Equation (15), is plotted in Figure 18. The relaxation time of the LA phonon exhibits critical slowing when approaching *T_ct_* from both sides of the transition. The value of *τ_o_*obtained from Equation (16) is 0.077 ps below *T_ct_* and 0.057 ps above *T_ct_*. This is about half of the value of KDP and TGS and is very similar to that obtained in PbHPO_4_ [75]. This suggests the weaker coupling of the LA phonon with the soft mode of the paraelectric host.

**Figure 19:**
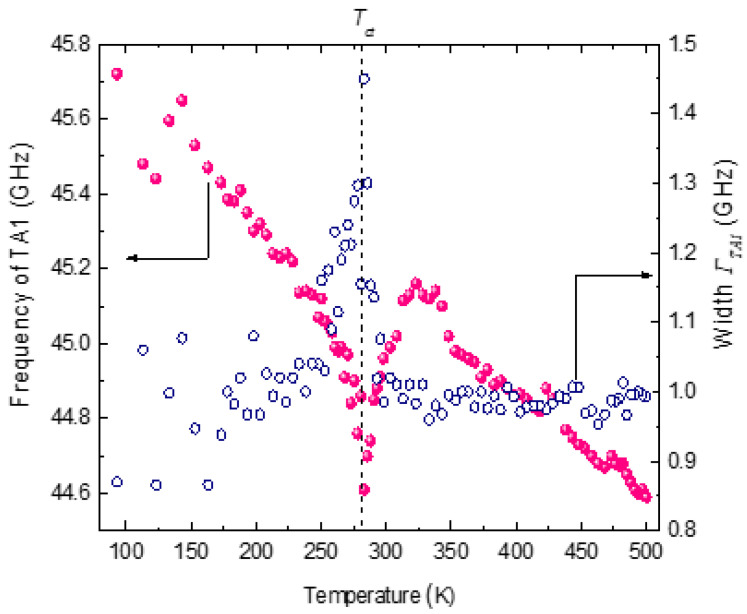
Temperature variation in thefrequency and the width of TA1 phonon mode. (Reprinted with kind permission from Ref. [83]. Copyright (2020) The Japan Society of Applied Physics).

**Figure 20:**
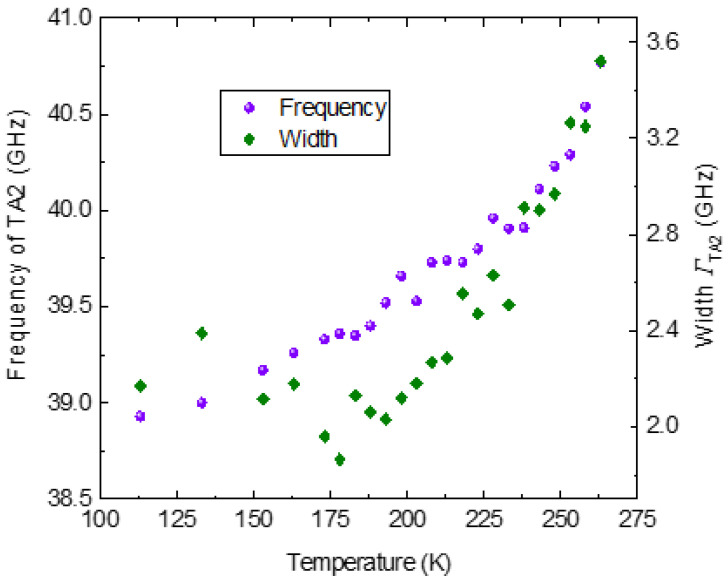
The frequency and the width of the TA2 phonon as a function of temperature.

The temperature dependence of the frequency and width of the TA1 and TA2 phonons are shown in Figure 19 and Figure 20. The frequency and the width of the TA1 phonon mode exhibit similar behavior as that of the LA phonon mode. However, a large scattering of the data observed in the width of the TA1 phonon mode limits further detailed discussion on its temperature dependence. The TA2 mode appears just near *T_ct_*. The experimental data, however, could be unambiguously fitted only below 265 K. The TA2 mode shows a significant softening with the decrease in temperature in the ferroelastic phase. In the Brillouin scattering study of phase transition in Cs_2_CdBr_4_, the splitting of TA mode into two components was observed on the phase transition from orthorhombic to monoclinic phase [88]. One of the components was purely TA mode (*C*_66_) corresponding to the parent orthorhombic phase, while the other was ascribed to the quasi-transverse mode γ_4_(b), propagating along the b axis of the crystal. The frequency of the γ_4_(b) mode was observed to decrease with decrease in temperature in the monoclinic phase. In SCT-0.03, the splitting of the TA phonon (TA1 and TA2 in the tetragonal phase) at *T_ct_* and the temperature dependence of TA2 mode in the ferroelastic phase appears to be quite similar to that observed in Cs_2_CdBr_4_. We therefore tentatively ascribe the TA2 phonon as the quasi-transverse mode propagating along the ***c*** axis of SCT-0.03 in the tetragonal phase. However, the correct description of TA2 mode requires detailed Brillouin light scattering studies in various polarization geometries.

## 5. Conclusions

Brillouin light scattering studies on the relaxation dynamics of disordered perovskite compounds have been reviewed. In PMT, the elastic constant *C*_11_ clearly reveals the existence of two distinct dynamical regimes of polarization characterized by the Burns temperature *T_B_* and the intermediate temperature T*. In the quasi-dynamic phase between *T_B_* and T*, the elastic constant obeys the mean field model and softens as *(T−T_o_)*^0.5^. The reduced softening of the elastic constant *C*_11_ and the increased attenuation in the quasi-static region below T*are attributed to the strong coupling between the local polarization and strain in PNRs. The temperature dependence of the relaxation time of the central peak was found to be non-Arrhenius. The observed critical slowing of the relaxation time between T* and *T_f_* is attributed to dipole flipping and the volume fluctuations of PNRs below *T_f_*. In the partially disordered PST, a sharp anomaly was observed in the frequency and the width of the LA phonon upon the transition from cubic relaxor to rhombohedral ferroelectric phase at 295 K. The calculated relaxation time of the LA phonon suggested the order–disorder nature of the structural phase transition. In 0.65PIN-0.35PT single crystal, anomalous behavior in the frequency and the width of the LA phonon was observed at cubic–tetragonal and tetragonal–rhombohedral phase transition temperatures. Two-component quasi-elastic (narrow and broad) central peaks were suggested to originate from two types of PNRs with different local structures. While the rhombohedral-type PNRs were pertinent to the parent relaxor PIN, the tetragonal-type PNRs areinduced by PT substitution. The broad central peak was suggested to associate with the relaxation of rhombohedral PNRs, while that of the narrow central peak to the relaxation of tetragonal PNRs. The relaxation time of the LA phonon was found to be qualitatively similar to that of the broad quasi-elastic central peak. The phase transition from cubic to tetragonal structure was suggested to originate from nucleation and growth of tetragonal PNRs. In SCT-0.03, the LA phonon showed critical anomalies at the structural phase transition temperature *T_ct_* = 280 K. The Landau–Khalatnikov-type asymmetry was observed in the width of the LA phonon. The relaxation time of the LA phonon showed a critical slowing in the vicinity of the transition.The observed acoustic anomaly was suggested to be due to the coupling between the LA phonon and the soft mode of SrTiO_3_. In the ferroelastic phase, the quasi-transverse TA (TA2) phonon mode was found to exhibit softening behavior with decrease in temperature. 

## Data Availability

The data sets generated during and/or analyzed during the current study are available from the corresponding author on request.
